# Bilateral Ptosis as the First Presentation of Guillain-Barre Syndrome

**Published:** 2016

**Authors:** Ahmad TALEBIAN, Babak SOLTANI, Motahhareh TALEBIAN

**Affiliations:** 1 Department of Pediatrics, Kashan University of Medical Sciences, Kashan, Iran.

**Keywords:** Guillain-Barre Syndrome, Children, Ptosis, Weakness

## Abstract

**Objective**

Guillain-Barre syndrome (GBS) is the most common cause of acute weakness in children. It has multiple variant forms with different presentations. A rare initial sign is ptosis. In this study, we present a 10-year-old girl with bilateral ptosis without opthalmoplegia followed by a weakness in extremities with a favourable response to intravenous immunoglobulin. Due to the patient’s initial eyelid levators, myasthenia gravis was ruled out by a Tensilon test and electrophysiological studies. Our report highlights the possibility of GBS as a cause of isolated ptosis, especially in cases without ophthalmoplegia.

## Introduction

Guillain-Barre syndrome is considered as an immune mediated acute inflammatory demyelinating polyradiculoneuropathy with rapid development of motor weakness. GBS has a wide range of variants with distinct features. The most common prototype is acute inflammatory demyelinating polyneuropathy (AIDP). The less common forms are axonal neuropathies which include, acute motor axonal neuropathy (AMAN), acute motor and sensory axonal neuropathy (AMSAN) and Miller Fisher syndrome (1). Approximately 50% of patients with GBS develop cranial neuropathy. However, facial or ocular muscles are affected first. The most common cranial nerve involvement is facial, whilst ocular muscle weakness is seen in 10% of patients. Moreover, eyelid levators are less affected common than other oculomotor muscles (2). Thus, at the onset of GBS, the presence of ptosis without ophthalmoplegia is rare. In this study, we report a case of GBS where the patient initially presented with bilateral ptosis without ophthalmoplegia or ataxia and, afterwards, developed ascending paralysis.

## Case Report

**Fig 1:**
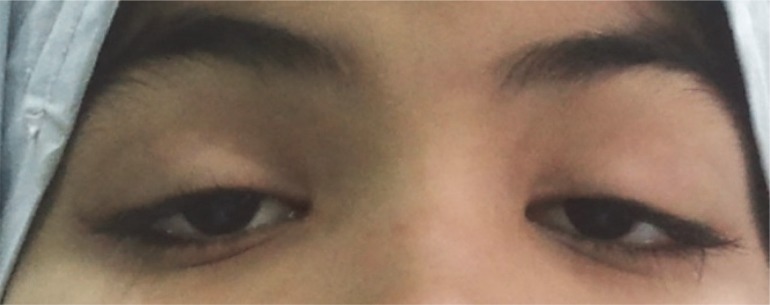
Bilateral ptosis at onset of disease

**Fig 2:**
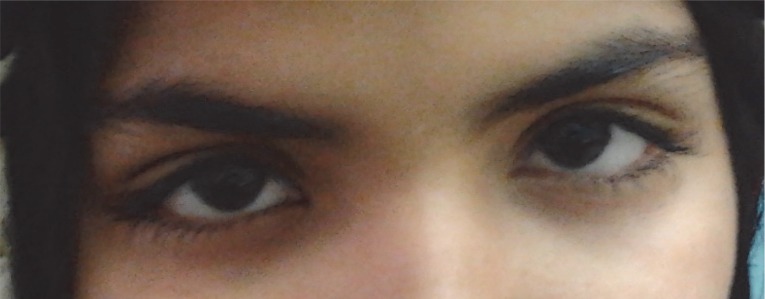
Resolution of ptosis a month after IVIG treatment

A previously healthy 10-year-old girl was presented with painless bilateral ptosis, which she had been suffering with for two days. At first, the ptosis only affected the left eye. However, following several hours, the right eye was also affected. The ptosis did not have any diurnal fluctuation and the patient was hospitalized. Two days later, she noticed a progressive weakness in her limbs, especially in the lower extremities. This meant that she was unable to walk. Her vaccinations were documented as up to date. She had no history of any flu-like diseases or recent gastrointestinal infections. Furthermore, a neurologic examination revealed that her mental status was normal. There was bilateral ptosis (Figure 1). Her eye movements were normal and her pupils were of normal size, symmetric and reactive to light. Other cranial nerves were also normal. The muscle forces of her upper and lower limbs were 4/5 and 3/5, respectively. Her tendon reflexes had significantly decreased in all extremities and no Babinsky signs were detected. She did not reveal any abnormalities in sensory, sphincteric and coordinating examinations. Additionally, her haematological and biochemical tests, such as liver, renal, thyroid functions, glucose and electrolytes, were normal. The Tensilon test was non-reactive. Furthermore, her brain and spinal MRIs were normal. There were no abnormalities in her cerebrospinal fluid (CSF), except for protein 102 mg/dl. Electrophysiological studies revealed acute polyneuropathy. Intravenous immunoglobulin (IVIG) was started for the patient (2g/kg), with a rapid resolution of muscle weakness. After a week, she walked without assistance. Three weeks later, the ptosis had significantly improved and in her follow up visits, it had disappeared completely (Figure 2).

## Discussion

Besides the typical acute inflammatory demyelinating polyneuropathy (AIDP) manifesting as areflexia and ascending paralysis peaking during a month, several known variants including axonal deficit, pure motor, sensory or autonomic involvement, Miller Fisher syndrome (MFS) and other local form are reported (3,4). For a patient with acute isolated ptosis, a complete neurologic examination is mandatory to distinguish whether the ptosis is isolated or not. Central nervous system (CNS) imaging, such as MRI, is suggested in cases where there are eye motion limitations, pupillary or visual disturbances, local weakness, other cranial nerve disorders and some atypical features, in addition to the ptosis. Neurologic consultation is usually necessary. After the exclusion of a mass lesion in brainstem and phenytoin poisoning, neuromuscular disorders must be considered (5). Having the first manifestation of cases with acute inflammatory neuropathy as ptosis without ophthalmoplegia is very rare. However, in MFS, ptosis with ophthalmoplegia is common (5). In our case, there were some findings which supported a diagnosis of GBS over MFS. The first was the absence of ophthalmoplegia and ataxia during the course of disease. The second finding that supported our diagnosis was that protein cell dissociation in CSF is uncommon in MFS. Acute isolated bilateral ptosis is more often observed in myasthenia gravis (MG) patients. Furthermore, cases of MG and GBS can later develop generalized paralysis. The diagnosis of MG was unlikely in our patient. This was due to the lack of response to the Tensilon test, lack of diurnal variety of symptoms and the results of the electrophysiological evaluations. One of the neuromuscular junction disorders, such as botulism, can also cause generalized paralysis and ptosis (6). In our patient, normal pupillary function led to the exclusion of botulism. More than 85% of cases with GBS with ophthalmoplegia and MFS have a positive anti-GQ1b IgG antibody. However, it is rarely positive among GBS patients without ophthalmoplegia (7). Stalpers et al. presented a GBS patient with isolated bilateral ptosis and ataxia (8). They had not achieved acute phase antiGQb1 IgG. Teng and Sung (9) reported a GBS patient with ptosis at the onset of disease. No anti-GQ1b IgG or CSF analysis was performed. In our study, serum antiGQ1b IgG was not measured. During an investigation of 33 GBS patients by Karimzadeh et al. (10), 24.3% had atypical presentations. These included: ptosis (3%), upper extremities weakness (3%), proximal weakness (9.1%), dysphagia (3%), headache (3%) and neck stiffness (3%). Furthermore, a descending weakness was seen in 15.2%, cranial neuropathy in 24.3% and facial nerve palsy in 9.1%. According to a normal brain MRI, a characteristic electrophysiological pattern compatible with acute polyneuropathy, normal pupillary function, no response to Tensilon test, albuminocytologic dissociation in CSF, pure ptosis without ophthalmoplegia and ataxia, no diurnal change in ptosis, ascending symmetric weakness of extremities in our patient, her diagnosis was consistent with GBS. The diagnosis of GBS should be kept in mind in patients with isolated ptosis and ptosis with ascending or descending paralysis of cranial or extremities. Furthermore, electrophysiological studies should be taken into consideration in cases with myasthenia gravis disorders. Furthermore, anti-GQ1b could differentiate some variants of GBS.
